# Models for identifying cognitive frailty in hospitalized older adults with chronic heart failure in China: a systematic review

**DOI:** 10.3389/fpubh.2026.1904724

**Published:** 2026-08-21

**Authors:** Yue Mou, Longyun Li, Qian Geng, Yao Li, Tingjia Zhang, Fang Cao, Yuanyuan Li, Ting Zhang

**Affiliations:** 1 Department of Health Humanities Research Center, Sichuan Vocational College of Health and Rehabilitation, Zigong, China; 2 North Sichuan Medical College, Nanchong, China; 3 The Second Affiliated Hospital of Army Medical University, Chongqing, China; 4 General Hospital of Western Theater Command, Chengdu, China

**Keywords:** chronic heart failure, cognitive frailty, diagnostic model, older adults, risk of bias, screening model, systematic review

## Abstract

**Objective:**

To systematically evaluate models for identifying cognitive frailty (CF) among hospitalized older adults with chronic heart failure (CHF) in China and assess their intended use, performance, and methodological quality.

**Methods:**

A systematic search was conducted in PubMed, Web of Science Core Collection, Embase, the Cochrane Library, China National Knowledge Infrastructure, Wanfang, VIP, and the Chinese Biomedical Literature Database from inception to March 31, 2026. Two reviewers independently screened the records according to the predefined eligibility criteria and extracted data using the Checklist for Critical Appraisal and Data Extraction for Systematic Reviews of Prediction Modeling Studies. For each included study, the operational definition of CF, the reported approach to dementia exclusion, and the timing of predictor and outcome assessment were rechecked. Models in which predictors and CF were assessed during the same hospitalization were classified as diagnostic or screening models for prevalent CF rather than prognostic models for future CF onset. Risk of bias and applicability were independently assessed using the Prediction Model Risk of Bias Assessment Tool.

**Results:**

Eight cross-sectional studies reporting 20 models were included. All were conducted among hospitalized older adults with CHF in China, and none predicted incident CF during follow-up. Development sample sizes ranged from 246 to 645, and CF prevalence ranged from 17.8% to 50.5%. Six studies performed internal validation, four performed external validation, and one reported no validation; three studies performed both internal and external validation. The area under the receiver operating characteristic curve in external validation ranged from 0.770 to 0.852. One study did not report calibration. The most frequently retained predictors were age, New York Heart Association functional classification, depression, nutritional status, and sleep-related factors. All studies had a high overall risk of bias, whereas applicability concerns were generally low.

**Conclusion:**

Existing evidence concerns concurrent identification of prevalent CF rather than prediction of future onset. Current models are not ready for routine clinical decision-making because of high risk of bias, incomplete reporting, limited external validation, and lack of clinical-impact evidence. Longitudinal model development, multicenter external validation, comprehensive calibration and clinical-utility assessment, and prospective impact evaluation are required before implementation.

**Systematic review registration:**

https://www.crd.york.ac.uk/PROSPERO/view/CRD420261413101, CRD420261413101.

## 1 Introduction

Chronic heart failure (CHF) is characterized by high morbidity, frequent rehospitalization, and high mortality, and represents the terminal stage of various cardiovascular diseases. It has become a major global public health concern (1, 2). With the acceleration of population aging, cognitive frailty (CF) among older adults with CHF has received increasing attention. CF is defined as a clinical syndrome characterized by the coexistence of mild cognitive impairment and physical frailty in the absence of dementia (3). Epidemiological evidence has shown that the prevalence of CF among older adults with CHF can reach 34.5% (4), which is substantially higher than the 2.1% reported among community-dwelling older adults (5). CF significantly increases the risk of adverse outcomes in older adults with CHF. Compared with patients without CF, those with CF have 1.55 times the risk of death and rehospitalization within 1 year (6), imposing a considerable burden on patients, families, and healthcare systems. As CF is considered potentially reversible and amenable to intervention (7), early identification and timely intervention are essential for improving patient outcomes. However, comprehensive assessment of both cognitive function and physical frailty may be time-consuming in routine clinical practice, while brief and practical tools for initial identification remain limited. In recent years, researchers in China have developed multivariable prediction models, often described in the primary studies as risk prediction models, to facilitate the identification of CF among older adults with CHF. Nevertheless, the existing studies are scattered, and a systematic synthesis and evaluation of these models is still lacking. Although models developed in other populations may provide methodological insights, their transportability to hospitalized older adults with CHF in China remains uncertain because differences in healthcare settings, patient case mix, predictor measurement, and the application of locally adapted cognitive and frailty assessment instruments, including education-adjusted cognitive cutoffs, may affect model performance. Restricting this review to studies conducted in China therefore enabled us to evaluate evidence generated within the Chinese healthcare and measurement context and to assess the applicability of these models to this population. Importantly, it remains unclear whether the existing models were intended to screen for or identify prevalent CF or to predict incident CF. Accordingly, this review aimed to systematically evaluate multivariable prediction models for CF among hospitalized older adults with CHF in China, clarify their intended clinical role, and assess their methodological quality, performance, applicability, and readiness for clinical use.

## 2 Methods

### 2.1 Study design and registration

This systematic review was reported in accordance with the Preferred Reporting Items for Systematic Reviews and Meta-Analyses (PRISMA) 2020 statement (8). The protocol was registered in PROSPERO (registration number: CRD420261413101).

### 2.2 Search strategy

A comprehensive literature search was conducted in PubMed, Web of Science Core Collection, Embase, the Cochrane Library, China National Knowledge Infrastructure, Wanfang Database, VIP Database, and the Chinese Biomedical Literature Database (CBM). The search period was from database inception to March 31, 2026. In addition to journal articles, eligible master's theses and doctoral dissertations identified through the Chinese database searches were also considered for inclusion, as some locally developed or validated prediction models may not have been published in peer-reviewed journals. Medical subject headings and free-text terms were combined, and the search strategy was adapted according to the requirements of each database.

The English search terms mainly included terms related to older adults, chronic heart failure, cognitive frailty, and prediction models, such as “aged,” “aging,” “geriatrics,” “older adult^*^,” “heart failure,” “chronic heart failure,” “congestive heart failure,” “cognitive dysfunction,” “frailty,” “cognitive frailty,” “mild cognitive impairment,” “risk prediction model,” “clinical prediction model,” “prognostic model,” “risk score,” “nomogram,” “machine learning,” and “deep learning.” The Chinese search terms included corresponding terms for older adults, heart failure, cognitive impairment, frailty, cognitive frailty, prediction, prediction model, risk assessment, risk factors, nomogram, and machine learning. The final searches were run on March 31, 2026. The database platform, complete search strategy, field restrictions, and number of records retrieved for each database are provided in Supplementary Table S1. No language restrictions were applied during the electronic searches; however, eligibility was restricted to studies published in Chinese or English during study selection.

### 2.3 Eligibility criteria

Studies were included if they met the following criteria: (1) the study population comprised Chinese older adults aged 60 years or above with a confirmed diagnosis of CHF; (2) CF was defined as the coexistence of objectively assessed physical frailty and cognitive impairment in the absence of dementia (use of a specific instrument for dementia exclusion, such as the Clinical Dementia Rating, was not required; incomplete reporting of the dementia-exclusion procedure did not by itself lead to exclusion but was considered when assessing risk of bias in the outcome domain); (3) cognitive function was assessed using standardized tools, such as the Montreal Cognitive Assessment or Mini-Mental State Examination, and physical frailty was assessed using tools such as the FRAIL scale or Fried frailty phenotype; (4) the study developed, validated, or updated a multivariable prediction model for CF; (5) the study design was observational; and (6) the model development process and performance indicators were reported.

Studies were excluded if they met any of the following criteria: (1) studies published in languages other than Chinese or English; (2) full text unavailable; (3) duplicate publications; (4) conference abstracts, reviews, clinical guidelines, case reports, or other non-original studies; and (5) studies that only explored risk factors without developing or validating a prediction model.

### 2.4 Study selection and data extraction

All retrieved records were imported into Note Express for reference management and duplicate removal. Study selection and data extraction were independently performed by two reviewers with evidence-based training. The reviewers first screened the titles and abstracts according to the predefined eligibility criteria and then assessed the full texts to determine final inclusion.

Data were extracted using a standardized form developed according to the Critical Appraisal and Data Extraction for Systematic Reviews of Prediction Modeling Studies (CHARMS) checklist (9). Extracted information included the first author, publication year, study design, study population, sample source, CF assessment tools, development sample size, internally derived test-set size, external validation sample size, prevalence of CF, number of candidate predictors, events per variable (EPV), final predictors, variable-selection methods, missing-data handling, model-development methods, model presentation, validation methods, and model-performance indicators, as applicable. We also extracted the timing of predictor and outcome assessment, the study-specific definition of CF, and the reported method used to exclude dementia. Based on the timing of predictor and outcome assessment, each model was classified as diagnostic/screening or prognostic. Models were classified as diagnostic or screening models when predictors and CF status were assessed during the same hospitalization or clinical encounter. Prognostic models were defined as those using predictors measured at baseline to estimate incident CF during a subsequent, explicitly defined follow-up period.

When a study evaluated multiple algorithms, each algorithm was treated as a separate model. Model-level data included the model role, algorithm or presentation, predictor set, dataset used for performance assessment, validation type, area under the receiver operating characteristic curve (AUC) with its 95% confidence interval, sensitivity, specificity, accuracy, F1 score, calibration measures, and decision-curve analysis, where available. Any discrepancies were resolved through discussion or consultation with a third reviewer. Study-specific CF assessment instruments and operational definitions, dementia-exclusion methods, and outcome-domain judgments are summarized in Supplementary Table S2, whereas detailed model-specific characteristics and performance estimates for all 20 models are presented in Supplementary Table S3.

### 2.5 Risk of bias and applicability assessment

The methodological quality and applicability of the included studies were assessed using the Prediction Model Risk of Bias Assessment Tool (PROBAST) (10). Risk of bias was evaluated across four domains: participants, predictors, outcome, and analysis. Applicability was assessed across three domains: participants, predictors, and outcome. Each domain was judged as low risk, high risk, or unclear risk for bias, and as low concern, high concern, or unclear concern for applicability. The overall risk of bias and applicability were determined according to PROBAST guidance. Two reviewers independently completed the assessment, and disagreements were resolved through discussion or consultation with a third reviewer.

### 2.6 Data synthesis

A narrative synthesis was conducted. The basic characteristics of the included studies, model-development and validation methods, model performance, final predictors, and PROBAST assessment results were summarized descriptively and presented in tables. Performance estimates were synthesized at the level of individual models. Development-sample performance, internal validation results—including bootstrap, cross-validation, and random-split test sets derived from the development dataset—and external validation results were reported separately because these settings provide methodologically distinct performance estimates. Statistical pooling was considered inappropriate when substantial differences existed in populations, outcome definitions, predictor sets, algorithms, or validation procedures.

## 3 Results

### 3.1 Study selection

**Figure 1:**
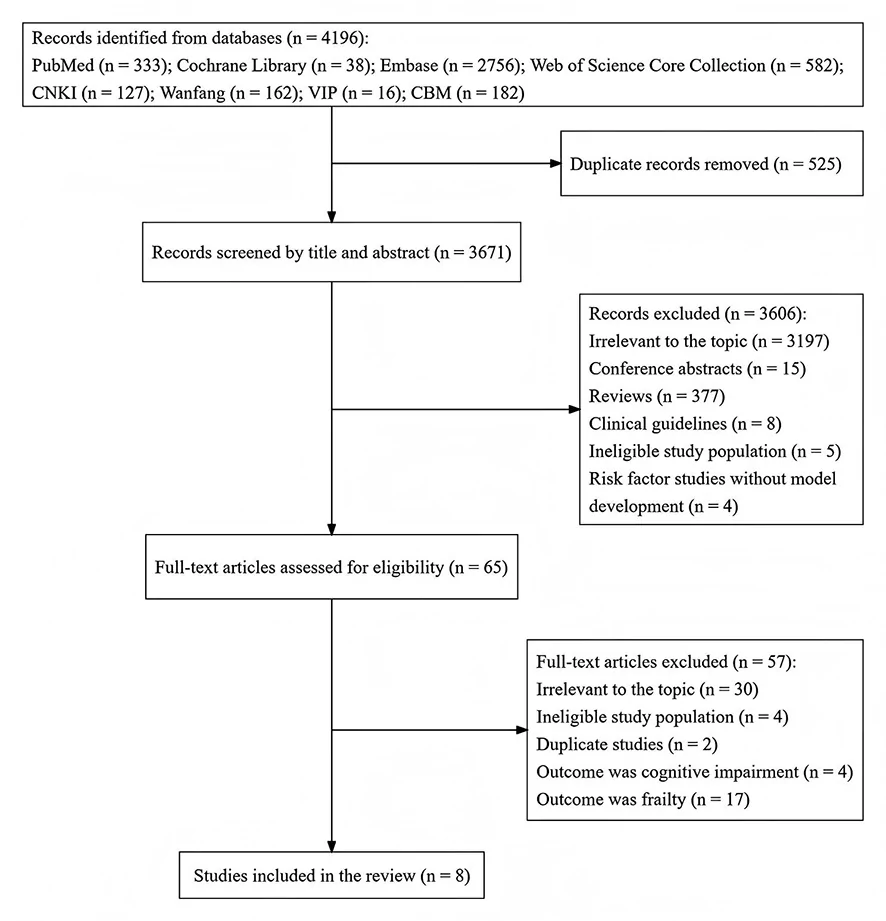
Flow diagram of study selection.

A total of 4,196 records were initially identified through database searches. After removing duplicates, 3,671 records remained for screening. Based on title and abstract screening, 3,606 records were excluded. The full texts of 65 potentially eligible studies were then assessed. Eight studies met the eligibility criteria and were ultimately included, comprising five Chinese-language studies and three English-language studies. Each included study investigated an outcome explicitly described by the original investigators as cognitive frailty and operationalized it using both a standardized cognitive assessment and a physical frailty assessment. The study-selection process is shown in Figure 1.

### 3.2 Characteristics of the included studies

The eight included studies were published between 2023 and 2026, and all were conducted in China. The study populations were hospitalized older adults aged 60 years or above with CHF. Four were multicenter studies (11–14). Cognitive function was most commonly assessed using the Montreal Cognitive Assessment (MoCA) (*n* = 5) (11, 13–16), followed by the Mini-Mental State Examination (MMSE) (*n* = 2) (12, 17) and the Montreal Cognitive Assessment-Basic (MoCA-BC) (*n* = 1) (18). Physical frailty was assessed using the Fried frailty phenotype (FP) in four studies (11–13, 16) and the FRAIL scale in four studies (14, 15, 17, 18). In addition, two studies used the Clinical Dementia Rating (CDR) to exclude dementia (11, 12). Other studies relied on explicit exclusion criteria, neurologist-confirmed diagnoses, or medical-record information to establish the absence of dementia, whereas two studies provided limited operational detail (Supplementary Table S2). The basic characteristics of the included studies are summarized in Table 1.

**Table 1:** Basic characteristics of the included studies (*n* = 8).

Study	Year	Population	Data source	Development sample, *n*	Internally derived test set, *n*	External validation sample, *n*	Assessment tools	Prevalence of CF, %	Candidate predictors, *n*	EPV
Liu (15)	2023	Hospitalized patients with CHF aged ≥60 years	Single center	327	—	—	MoCA + FRAIL scale	50.5	18	9.17
Jiang (11)	2023	Hospitalized patients with CHF aged ≥60 years	Multicenter	261	—	132	MoCA + CDR + FP	34.48	10	9.0
Luo (12)	2024	Hospitalized patients with CHF aged ≥60 years	Multicenter	313	—	138	MMSE + CDR + FP	30.7	8	12.0
Chen et al. (13)	2025	Hospitalized patients with CHF aged ≥60 years	Multicenter	424	183	—	MoCA + FP	48.3	9	22.78
Li et al. (17)	2025	Hospitalized patients with CHF aged ≥60 years	Single center	435	—	187	MMSE + FRAIL scale	31.03	10	13.5
Liu et al. (14)	2026	Hospitalized patients with CHF aged ≥60 years	Multicenter	390	—	80	MoCA + FRAIL scale	46.9	6	30.5
Wang et al. (16)	2026	Hospitalized patients with CHF aged ≥60 years	Single center	246	—	—	MoCA + FP	35.0^*^	11	7.82
Gou et al. (18)	2026	Hospitalized patients with CHF aged ≥60 years	Single center	645	162	—	MoCA-BC + FRAIL scale	17.8	38	3.03

“—”, not reported; MoCA, Montreal Cognitive Assessment; MoCA-BC, Montreal Cognitive Assessment-Basic; MMSE, Mini-Mental State Examination; CDR, Clinical Dementia Rating; FP, Fried frailty phenotype; EPV, events per variable; EPV was calculated as the number of participants with cognitive frailty in the development sample divided by the number of candidate predictors. CF prevalence refers to the study population reported in each original study. ^*^CF prevalence was calculated as the number of CF cases divided by the total sample size because it was not directly reported in the original study.

All eight studies were cross-sectional. Predictors and CF status were assessed during the same hospitalization or clinical encounter; therefore, all included models were classified as diagnostic or screening models for prevalent CF, and none were classified as prognostic models for incident CF. Development sample sizes ranged from 246 to 645, and the prevalence of CF ranged from 17.8% to 50.5%.

### 3.3 Model development, validation, and performance

The eight included studies reported a total of 20 models for identifying CF in older adults with CHF. Six studies used univariate analysis for preliminary variable screening (12–17), whereas two studies used least absolute shrinkage and selection operator (LASSO) regression for variable selection (11, 18). Five studies reported using complete-case analysis or direct deletion to handle missing data (11, 12, 14–16).

Regarding modeling approaches, six studies developed logistic-regression models and presented them as nomograms (11, 12, 14–17). Chen et al. (13) compared eight algorithms, including neural network, linear discriminant analysis (LDA), k-nearest neighbors, support vector machine, naive Bayes, logistic regression, decision tree, and random forest. Gou et al. (18) compared six algorithms, including categorical boosting (CatBoost), extreme gradient boosting (XGBoost), logistic regression, support vector machine, random forest, and multilayer perceptron.

Six studies reported internal validation (11–13, 15, 17, 18), four studies reported external validation (11, 12, 14, 17), and one study did not report model validation (16). Three studies reported both internal and external validation (11, 12, 17). In the four studies with external validation, AUC values ranged from 0.770 to 0.852 (11, 12, 14, 17). Apparent performance estimates from development samples, internal validation estimates, and external validation estimates were reported separately because they were not directly comparable. Chen et al. (13) and Gou et al. (18) identified LDA and CatBoost as their preferred models, respectively; both comparisons were based on internally derived test sets. Chen et al. (13) did not report calibration. Table 2 provides a concise study-level overview of model development, validation, and performance, whereas Supplementary Table S3 presents detailed model-specific characteristics and performance estimates for all 20 models.

**Table 2:** Study-level development, validation, and performance of models for identifying cognitive frailty (*n* = 8).

Study	Variable selection method	Missing data handling	Modeling method	Model presentation	Validation method	Discrimination	Calibration assessment	Sensitivity	Specificity
Liu (15)	Univariate analysis	Direct deletion	LR	Nomogram	IV (bootstrap)	Development sample: AUC = 0.804; IV: AUC = 0.788	H-L test, brier score, calibration curve	Development sample: 0.772	Development sample: 0.703
Jiang (11)	LASSO regression	Direct deletion	LR	Nomogram	IV (bootstrap) + EV	Development sample: AUC = 0.854; EV: AUC = 0.852	Calibration curve	EV: 0.872	EV: 0.694
Luo (12)	Univariate analysis	Direct deletion	LR	Nomogram	IV (bootstrap) + EV	Development sample: AUC = 0.949; EV: AUC = 0.799	H-L test, brier score, calibration curve	Development sample: 0.896; EV: 0.792	Development sample: 0.876; EV: 0.689
Chen et al. (13)	Univariate analysis and stepwise LR	—	NN, LDA, KNN, SVM, NB, LR, DT, RF	—	IV (random split)	Internally derived test set: AUC range of all models: 0.820–0.901	—	Internally derived test set: LDA = 0.778; all models = 0.700–0.778	—
Li et al. (17)	Univariate analysis and stepwise LR	—	LR	Nomogram	IV + EV	IV: AUC = 0.867; EV: AUC = 0.848	H-L test, calibration curve	IV: 0.859; EV: 0.839	IV: 0.760; EV: 0.794
Liu et al. (14)	Univariate analysis	Direct deletion	LR	Nomogram	EV	Development sample: AUC = 0.807; EV: AUC = 0.770	H-L test, calibration curve	—	—
Wang et al. (16)	Univariate analysis	Direct deletion	LR	Nomogram	—	Development sample: AUC = 0.846	H-L test, calibration curve	Development sample: 0.779	Development sample: 0.800
Gou et al. (18)	LASSO regression	—	CatBoost, XGBoost, LR, SVM, RF, MLP	—	IV (random split, 5-fold cross-validation, bootstrap)	Internally derived test set: AUC range of all models: 0.890–0.916	Brier score	Internally derived test set: CatBoost = 0.922; all models = 0.866–0.922	Internally derived test set: CatBoost = 0.805; all models = 0.773–0.825

“—”, not reported; AUC, area under the receiver operating characteristic curve; IV, internal validation; EV, external validation; H-L test, Hosmer–Lemeshow goodness-of-fit test; LR, logistic regression; LASSO, least absolute shrinkage and selection operator; NN, neural network; LDA, linear discriminant analysis; KNN, k-nearest neighbors; SVM, support vector machine; NB, naive Bayes; DT, decision tree; RF, random forest; MLP, multilayer perceptron; XGBoost, extreme gradient boosting; CatBoost, categorical boosting.

### 3.4 Final predictors included in the models

The final predictors included in the models were grouped into three categories: sociodemographic factors, disease-related and function-related factors, and psychological, behavioral, and social support factors. Sociodemographic factors included age, sex, educational level, marital status, and living status. Disease-related and function-related factors included New York Heart Association (NYHA) functional classification, nutritional status, chewing ability, body mass index, multimorbidity, duration of CHF, left ventricular ejection fraction, history of cardiac stent implantation, medication use, activities of daily living, and history of hospitalization due to CHF. Psychological, behavioral, and social support factors included depression, loneliness, sleep-related factors, intellectual activity, weekly exercise frequency, and social support.

To improve comparability across studies, conceptually similar predictors were grouped under broader categories for descriptive synthesis. For example, malnutrition and nutritional status were classified under nutritional status, whereas sleep status, sleep duration, and sleep disturbance were grouped as sleep-related factors. These variables were grouped for summary purposes but were not considered identical measurements. The five most frequently reported predictors were age (*n* = 7) (11–14, 16–18), NYHA functional classification (*n* = 7) (11, 12, 14–18), depression (*n* = 5) (11, 12, 16–18), nutritional status (*n* = 5) (11, 13–15, 18), and sleep-related factors (*n* = 5) (11, 12, 14, 15, 18).

Several retained predictors were conceptually close to components of the physical frailty outcome. For example, exercise frequency may overlap with the low-physical-activity component of the Fried frailty phenotype, whereas activities of daily living and nutritional status may overlap with mobility- or weight-loss-related components of the frailty instruments used in the included studies. This potential construct overlap was considered in the revised PROBAST assessment of the predictors domain. The final predictors included in each study are summarized in Table 3.

**Table 3:** Summary of final predictors included in the models (*n* = 8).

Study	Final predictors
Liu (15)	NYHA functional classification, chewing ability, nutritional status, loneliness, sleep status
Jiang (11)	Age, educational level, NYHA functional classification, LVEF, sleep duration, nutritional status, depression, intellectual activity, social support
Luo (12)	Age, educational level, sleep duration, exercise frequency, NYHA functional classification, duration of CHF, history of cardiac stent implantation, depression
Chen et al. (13)	Age, marital status, educational level, body mass index, multimorbidity, nutritional status, medication use, weekly exercise frequency, living status
Li et al. (17)	Age, regular exercise, ADL, NYHA functional classification, depression
Liu et al. (14)	Age, NYHA functional classification, chewing ability, nutritional status, loneliness, sleep status
Wang et al. (16)	Age, depression, NYHA functional classification, exercise frequency, weekly intellectual activity
Gou et al. (18)	Age, sex, educational level, marital status, duration of CHF, history of hospitalization due to CHF, NYHA functional classification, malnutrition, ADL, sleep disturbance, depression

ADL, activities of daily living; LVEF, left ventricular ejection fraction.

### 3.5 Risk of bias and applicability assessment

The PROBAST assessment showed that all eight studies had a high overall risk of bias, whereas concerns regarding applicability were generally low. In the participants domain, one study was rated as having a high risk of bias because it was retrospective and included only patients with complete clinical data, which may have introduced selection bias (16), whereas the remaining studies were rated as low risk in this domain.

In the predictors domain, the studies were rated as having either unclear or high risk of bias. Concerns arose when candidate predictors were insufficiently defined, when it was unclear whether predictor assessment was performed without knowledge of outcome status, or when predictors conceptually overlapped with components of the physical frailty outcome. In the outcome domain, the studies were rated as having either low or unclear risk of bias. Unclear judgments arose when the operational procedures used to exclude dementia were incompletely reported, resulting in uncertainty regarding potential outcome misclassification.

In the analysis domain, all studies were rated as having a high risk of bias. Common methodological limitations included an insufficient effective sample size in some studies, data-driven predictor selection, incomplete or inappropriate handling of missing data, reliance on random-split internal validation, inadequate accounting for overfitting and optimism, and incomplete reporting of calibration. The study-level PROBAST judgments are presented in Table 4, and the corresponding study-specific rationales are provided in Supplementary Table S4.

**Table 4:** Risk of bias and applicability assessment of the included studies (*n* = 8).

Study	Risk of bia				Applicabilit			Overall	
	Participants	Predictors	Outcome	Analysis	Participants	Predictors	Outcome	Risk of bias	Applicability
Liu (15)	+	?	+	–	+	+	+	–	+
Jiang (11)	+	?	+	–	+	+	+	–	+
Luo (12)	+	–	+	–	+	+	+	–	+
Chen et al. (13)	+	–	?	–	+	+	+	–	+
Li et al. (17)	+	–	+	–	+	+	+	–	+
Liu et al. (14)	+	?	+	–	+	+	+	–	+
Wang et al. (16)	–	–	?	–	+	+	+	–	+
Gou et al. (18)	+	–	+	–	+	+	+	–	+

“+” indicates low risk of bias or low concern regarding applicability; “–” indicates high risk of bias or high concern regarding applicability; “?” indicates unclear risk of bias or unclear concern regarding applicability.

## 4 Discussion

### 4.1 Current evidence on models for identifying cognitive frailty in older adults with chronic heart failure in China

This review identified eight cross-sectional studies evaluating 20 multivariable models for CF among hospitalized older adults with CHF in China. In every study, predictors and CF status were assessed during the same hospitalization or clinical encounter. The available evidence therefore concerns the concurrent identification of prevalent CF rather than the prediction of future CF onset. This distinction is clinically important because discrimination estimated from cross-sectional data reflects classification at the time of assessment and should not be interpreted as an estimate of subsequent CF risk. The wide variation in CF prevalence, ranging from 17.8% to 50.5%, may be related to differences in study populations, assessment instruments, and diagnostic thresholds.

Performance estimates differed according to whether they were derived from the development sample, internal validation, or external validation. External validation yielded AUC values ranging from 0.770 to 0.852, whereas higher values were often reported in development samples or internal validation. These estimates are not directly comparable because apparent performance is more susceptible to optimism, while random-split test sets are still drawn from the same source population as the training data (10). Together with the high overall risk of bias, the available performance estimates support further model evaluation rather than routine clinical implementation.

Because all included studies were conducted among hospitalized older adults with CHF in China, the findings primarily reflect this specific population and clinical setting. The transportability of these models to outpatient or community populations, populations in other countries, and patients with different CHF phenotypes remains uncertain and requires independent external validation.

Most regression-based models were presented as nomograms, which may facilitate visual probability estimation but still require manual scoring and interpretation. Conversion into web-based calculators, mobile applications, or electronic medical record–embedded tools could improve usability; however, such implementation should occur only after adequate external validation, assessment of calibration and clinical utility, and prospective evaluation of clinical impact. At present, the models should be regarded as research or preliminary screening tools rather than stand-alone clinical decision-making instruments.

### 4.2 Methodological limitations and risk of bias in existing models

The PROBAST assessment indicated that all included studies had a high overall risk of bias. Differences in reported performance probably reflected both clinical and methodological heterogeneity. The studies used different cognitive and physical frailty assessment instruments, including MoCA, MoCA-BC, MMSE, FRAIL, and the Fried frailty phenotype, and varied in their approaches to dementia exclusion, including the use of CDR in some studies. Differences in education-adjusted cognitive cutoffs and definitions of physical frailty were also observed. Variation in CF prevalence, disease severity, candidate-predictor sets, variable-selection procedures, effective sample size, and validation methods may have further influenced the reported AUC values. In addition, predictors such as exercise frequency, activities of daily living, and nutritional status may conceptually overlap with components of the physical frailty outcome. If a predictor forms part of the definition or assessment of the physical frailty outcome, such overlap may introduce incorporation bias, leading to overestimation of the association between the predictor and the outcome and optimistic estimates of model performance (10).

Methodological weaknesses were common. Conventional EPV calculations suggested sparse outcome information in several studies, increasing the potential for overfitting and optimistic performance estimates (10). Future studies should use formal sample-size methods that consider outcome prevalence, anticipated model fit, the number of candidate predictor parameters, and expected optimism (19). Complete-case analysis or direct deletion may reduce precision and introduce selection bias when data are not missing completely at random; multiple imputation with transparent reporting is generally preferable (20). Limitations in variable selection were also identified. Most studies relied on univariate screening to select candidate predictors. Such data-driven selection may omit clinically important predictors that are not statistically significant in univariate analyses and may produce unstable predictor sets. Future studies should prespecify candidate predictors based on clinical knowledge and prior evidence and consider appropriate penalization methods, such as LASSO, when necessary (21).

Simple random splitting can be inefficient and may produce unstable performance estimates, particularly in modest datasets. Bootstrap validation or cross-validation is generally preferable for internal validation; however, neither can substitute for independent external validation (22). Although all included studies reported discrimination, one study did not report calibration (13), and some studies did not report sensitivity, specificity, or both. Future studies should report calibration using calibration plots together with quantitative measures, such as the calibration intercept and calibration slope, and should transparently report measures of clinical utility, such as decision-curve analysis, when clinical utility is evaluated, in accordance with TRIPOD+AI (23).

Two studies compared multiple machine-learning algorithms (13, 18). Chen et al. (13) identified LDA as the preferred model, whereas Gou et al. (18) reported the numerically highest AUC for CatBoost. However, both comparisons were based on internally derived test sets and lacked external validation. Therefore, the current evidence does not demonstrate that machine-learning approaches outperform logistic regression. Moreover, model evaluation should not focus on discrimination alone. The clinical value of machine-learning models also depends on whether clinicians can understand how individual predictions are generated and which factors contribute to them, whether model development is transparently reported and the final model is fully specified and reproducible, and whether the models can be feasibly integrated into routine clinical workflows. Future comparisons should therefore evaluate calibration, clinical utility, interpretability, transparency, and the feasibility of implementation in clinical practice in addition to discrimination.

### 4.3 Predictors and clinical implications

The most frequently reported predictors were age and NYHA functional classification, each included in seven studies, followed by depression, nutritional status, and sleep-related factors, each included in five studies. These variables may recur because they capture different but interrelated dimensions of vulnerability in older adults with CHF. Older age may reflect declining physiological and cognitive reserve, whereas a higher NYHA functional class may indicate greater symptom burden and functional limitation (24). Poor nutritional status may be accompanied by sarcopenia and reduced physiological reserve (25), while depression and sleep problems may be associated with increased cognitive and functional vulnerability (24, 26). Together, these factors may be associated with a greater likelihood of coexisting cognitive impairment and physical frailty. However, frequency of inclusion does not establish causality, predictor stability, or incremental predictive value.

These routinely available predictors may help clinicians identify patients who warrant further standardized cognitive and physical frailty assessment. Particular attention may be given to older patients with advanced NYHA functional class, depressive symptoms, nutritional risk, or sleep problems. However, these factors should not be used individually as diagnostic criteria or stand-alone clinical decision-making indicators. Future studies should evaluate their stability and incremental value beyond a transparent baseline model and determine whether their inclusion improves calibration and clinical utility.

### 4.4 Future research and clinical implementation

Future models should consider and formally evaluate routinely available objective measures relevant to CHF while maintaining parsimony. Measures previously examined in relation to cognitive dysfunction in older adults with heart failure include NT-proBNP, inflammatory biomarkers, hematologic indices, and selected echocardiographic parameters (27), while cardiac troponins and renal function measures may also warrant evaluation as additional CHF-related candidate predictors. However, their predictive value for CF should not be assumed solely on the basis of biological or clinical plausibility. Their incremental contribution beyond readily available demographic, clinical, functional, and psychosocial predictors should be quantified using appropriate measures of model performance and clinical utility.

To develop genuine prognostic models, future studies should measure predictors at a clearly defined baseline and ascertain incident CF during follow-up according to a prespecified prediction horizon. Time-to-event methods may be appropriate when follow-up duration varies or censoring occurs, whereas dynamic models may update estimated risk using repeated measurements of symptoms, functional status, or biomarkers. Multicenter external validation should involve different geographical regions, hospitals, outpatient clinics, and community settings and should assess model transportability across heart failure phenotypes with preserved, mildly reduced, and reduced ejection fraction.

Clinical implementation requires more than converting a nomogram into a web-based calculator, mobile application, or electronic medical record alert. Before routine use, a model should undergo independent external validation, recalibration where necessary, assessment of discrimination, calibration, and clinical utility, evaluation of fairness and usability, prospective clinical-impact studies, and implementation research. Future studies should follow the TRIPOD+AI reporting guidance (23), which applies to both regression-based and machine-learning prediction models, and future methodological appraisals should use PROBAST+AI (28) where appropriate.

## 5 Limitations

This review has several limitations. First, eligibility was restricted to studies published in Chinese or English, which may have introduced language bias and resulted in the omission of relevant studies published in other languages. Second, only eight studies were included, and all were cross-sectional studies conducted among hospitalized older adults with CHF in China. Therefore, the findings may not be generalizable to outpatient or community-dwelling populations or populations in other countries, and the included models cannot be interpreted as predicting future CF. In addition, because the included studies did not report model performance separately for heart failure with preserved, mildly reduced, and reduced ejection fraction, the transportability of the models across HF phenotypes remains uncertain. Third, substantial heterogeneity existed in the cognitive and physical frailty assessment tools used, the candidate predictors considered, model-development methods, and validation procedures; therefore, quantitative pooling of model performance estimates was not considered appropriate. Fourth, reporting of dementia exclusion, missing-data handling, calibration, complete model specifications, and validation procedures was incomplete in several primary studies, limiting detailed methodological appraisal and model reproducibility. Finally, the small evidence base limited formal assessment of publication bias and prevented reliable comparisons between modeling approaches.

## 6 Conclusion

This systematic review identified eight cross-sectional studies evaluating 20 models for the concurrent identification of CF among hospitalized older adults with CHF in China. These models should be regarded as diagnostic or screening models for prevalent CF rather than prognostic models for future CF. Although AUC values from external validation ranged from 0.770 to 0.852, all included studies had a high overall risk of bias, and evidence regarding calibration, transportability, and clinical impact remained limited. Age, NYHA functional classification, depression, nutritional status, and sleep-related factors were frequently retained as predictors, but their stability and incremental predictive value require formal evaluation in future studies. Existing models are not ready for routine clinical decision-making. Longitudinal studies for prognostic model development, rigorous multicenter external validation, complete calibration reporting, clinical-impact evaluation, and transparent reporting in accordance with TRIPOD+AI are needed before clinical implementation.

## Data Availability

The original contributions presented in the study are included in the article/Supplementary material, further inquiries can be directed to the corresponding author.
