# Supplementary material for: Models for identifying cognitive frailty in hospitalized older adults with chronic heart failure in China: a systematic review
Source: Front Public Health. 2026 Aug 21;14:1904724. doi: 10.3389/fpubh.2026.1904724 (PMC13541494; doi:10.3389/fpubh.2026.1904724)
Supplement: Supplementary file 1 [file Table_1.docx]

**Supplementary Material**

**Supplementary Table S1. Detailed search strategies and retrieval counts for each database**

| **Database** | **Platform** | **Coverage** | **Date last searched** | **Records retrieved** |
| --- | --- | --- | --- | --- |
| PubMed | National Library of Medicine | From inception to March 31, 2026 | March 31, 2026 | 333 |
| Cochrane Library | Wiley | From inception to March 31, 2026 | March 31, 2026 | 38 |
| Embase | Elsevier | From inception to March 31, 2026 | March 31, 2026 | 2,756 |
| Web of Science Core Collection | Clarivate | From inception to March 31, 2026 | March 31, 2026 | 582 |
| China National Knowledge Infrastructure (CNKI) | CNKI | From inception to March 31, 2026 | March 31, 2026 | 127 |
| Wanfang Data | Wanfang Data | From inception to March 31, 2026 | March 31, 2026 | 162 |
| VIP Database | VIP | From inception to March 31, 2026 | March 31, 2026 | 16 |
| Chinese Biomedical Literature Database (CBM) | SinoMed | From inception to March 31, 2026 | March 31, 2026 | 182 |
| Total | All eight databases | From inception to March 31, 2026 | March 31, 2026 | 4,196 |

No language restriction was applied during the electronic searches. Eligibility was restricted to studies published in Chinese or English during study selection.

| **Database** | **Search strategy** |
| --- | --- |
| **PubMed** | #1 Aged[Mesh] OR Aging[Mesh] OR Geriatrics[Mesh] OR aged[Title/Abstract] OR aging[Title/Abstract] OR elderly[Title/Abstract] OR older adult*[Title/Abstract] OR "older people"[Title/Abstract] OR senior*[Title/Abstract] OR geriatric*[Title/Abstract] #2 "Heart Failure"[Mesh] OR "heart failure"[Title/Abstract] OR "chronic heart failure"[Title/Abstract] OR "chronic cardiac failure"[Title/Abstract] OR "chronic myocardial failure"[Title/Abstract] OR "congestive heart failure"[Title/Abstract] #3 "Cognitive Dysfunction"[Mesh] OR "Frailty"[Mesh] OR "cognitive frailty"[Title/Abstract] OR "mild cognitive dysfunction"[Title/Abstract] OR mild cognitive decline*[Title/Abstract] OR mild cognitive impairment*[Title/Abstract] OR mild cognitive disorder*[Title/Abstract] OR "frail syndrome"[Title/Abstract] #4 "Prognosis"[Mesh] OR "Risk Assessment"[Mesh] OR "Forecasting"[Mesh] OR predict*[Title/Abstract] OR "prognostic model"[Title/Abstract] OR "risk prediction model"[Title/Abstract] OR "clinical prediction model"[Title/Abstract] OR "risk score"[Title/Abstract] OR "risk factor"[Title/Abstract] OR nomogram[Title/Abstract] OR "machine learning"[Title/Abstract] OR "deep learning"[Title/Abstract] #5 #1 AND #2 AND #3 AND #4 |
| **Cochrane Library** | #1 MeSH descriptor: [Aged] explode all trees OR MeSH descriptor: [Aging] explode all trees OR MeSH descriptor: [Geriatrics] explode all trees OR aged:ti,ab,kw OR aging:ti,ab,kw OR elderly:ti,ab,kw OR "older adult*":ti,ab,kw OR "older people":ti,ab,kw OR senior*:ti,ab,kw OR geriatric*:ti,ab,kw #2 MeSH descriptor: [Heart Failure] explode all trees OR "heart failure":ti,ab,kw OR "chronic heart failure":ti,ab,kw OR "chronic cardiac failure":ti,ab,kw OR "chronic myocardial failure":ti,ab,kw OR "congestive heart failure":ti,ab,kw #3 MeSH descriptor: [Cognitive Dysfunction] explode all trees OR MeSH descriptor: [Frailty] explode all trees OR "cognitive frailty":ti,ab,kw OR "mild cognitive dysfunction":ti,ab,kw OR "mild cognitive decline*":ti,ab,kw OR "mild cognitive impairment*":ti,ab,kw OR "mild cognitive disorder*":ti,ab,kw OR "frail syndrome":ti,ab,kw #4 MeSH descriptor: [Prognosis] explode all trees OR MeSH descriptor: [Risk Assessment] explode all trees OR MeSH descriptor: [Forecasting] explode all trees OR predict*:ti,ab,kw OR "prognostic model":ti,ab,kw OR "risk prediction model":ti,ab,kw OR "clinical prediction model":ti,ab,kw OR "risk score":ti,ab,kw OR "risk factor":ti,ab,kw OR nomogram:ti,ab,kw OR "machine learning":ti,ab,kw OR "deep learning":ti,ab,kw #5 #1 AND #2 AND #3 AND #4 |
| **Embase** | #1 'aged'/exp OR 'aging'/exp OR 'geriatrics'/exp OR aged:ab,ti OR aging:ab,ti OR elderly:ab,ti OR 'older adult':ab,ti OR 'older people':ab,ti OR senior*:ab,ti OR geriatric*:ab,ti #2 'heart failure'/exp OR 'heart failure':ab,ti OR 'chronic heart failure':ab,ti OR 'chronic cardiac failure':ab,ti OR 'chronic myocardial failure':ab,ti OR 'congestive heart failure':ab,ti #3 'cognitive dysfunction'/exp OR 'frailty'/exp OR 'cognitive frailty':ab,ti OR 'mild cognitive dysfunction':ab,ti OR 'mild cognitive decline':ab,ti OR 'mild cognitive impairment':ab,ti OR 'mild cognitive disorder':ab,ti OR 'frail syndrome':ab,ti #4 'prognosis'/exp OR 'risk assessment'/exp OR 'forecasting'/exp OR predict*:ab,ti OR 'prognostic model':ab,ti OR 'risk prediction model':ab,ti OR 'clinical prediction model':ab,ti OR 'risk score':ab,ti OR 'risk factor':ab,ti OR nomogram:ab,ti OR 'machine learning':ab,ti OR 'deep learning':ab,ti #5 #1 AND #2 AND #3 AND #4 |
| **Web of Science Core Collection** | #1 TS=(aged OR aging OR elderly OR "older adult*" OR "older people" OR senior* OR geriatric*) #2 TS=("heart failure" OR "chronic heart failure" OR "chronic cardiac failure" OR "chronic myocardial failure" OR "congestive heart failure") #3 TS=("cognitive dysfunction" OR frailty OR "cognitive frailty" OR "mild cognitive dysfunction" OR "mild cognitive decline*" OR "mild cognitive impairment*" OR "mild cognitive disorder*" OR "frail syndrome") #4 TS=(predict* OR "prognostic model" OR "risk prediction model" OR "clinical prediction model" OR "risk score" OR "risk factor" OR nomogram OR "machine learning" OR "deep learning") #5 #1 AND #2 AND #3 AND #4 |
| **China National Knowledge Infrastructure (CNKI)** | TKA=(老年人+老人) AND TKA=(心力衰竭+心衰+慢性心力衰竭+慢性心衰+慢性心功能不全+慢性充血性心力衰竭) AND TKA=(认知障碍+认知功能障碍+轻度认知功能障碍+轻度认知功能损害+轻度认知功能损伤+轻度认知功能减退+衰弱+躯体衰弱+虚弱+脆弱+认知衰弱) AND TKA=(预测+预测模型+预测因子+预测因素+危险因素+风险预测+风险评估+列线图+机器学习) |
| **Wanfang Data** | 主题:("老年人" OR "老人") AND 主题:("心力衰竭" OR "心衰" OR "慢性心力衰竭" OR "慢性心衰" OR "慢性心功能不全" OR "慢性充血性心力衰竭") AND 主题:("认知障碍" OR "认知功能障碍" OR "轻度认知功能障碍" OR "轻度认知功能损害" OR "轻度认知功能损伤" OR "轻度认知功能减退" OR "衰弱" OR "躯体衰弱" OR "虚弱" OR "脆弱" OR "认知衰弱") AND 主题:("预测" OR "预测模型" OR "预测因子" OR "预测因素" OR "危险因素" OR "风险预测" OR "风险评估" OR "列线图" OR "机器学习") |
| **VIP Database** | M=(老年人+老人) AND M=(心力衰竭+心衰+慢性心力衰竭+慢性心衰+慢性心功能不全+慢性充血性心力衰竭) AND M=(认知障碍+认知功能障碍+轻度认知功能障碍+轻度认知功能损害+轻度认知功能损伤+轻度认知功能减退+衰弱+躯体衰弱+虚弱+脆弱+认知衰弱) AND M=(预测+预测模型+预测因子+预测因素+危险因素+风险预测+风险评估+列线图+机器学习) |
| **Chinese Biomedical Literature Database (CBM/SinoMed)** | #1 "老年人"[不加权:扩展] OR "老年人, 80以上"[不加权:扩展] #2 "老年人"[全部字段:智能] OR "老人"[全部字段:智能] #3 #1 OR #2 #4 "心力衰竭"[不加权:扩展] #5 "心力衰竭"[全部字段:智能] OR "心衰"[全部字段] OR "慢性心力衰竭"[全部字段:智能] OR "慢性心衰"[全部字段:智能] OR "慢性心功能不全"[全部字段:智能] OR "慢性充血性心力衰竭"[全部字段:智能] #6 #4 OR #5 #7 "认知功能障碍"[不加权:扩展] OR "认知障碍"[不加权:扩展] OR "衰弱"[不加权:扩展] #8 "认知障碍"[全部字段:智能] OR "认知功能障碍"[全部字段:智能] OR "轻度认知功能障碍"[全部字段:智能] OR "轻度认知功能损害"[全部字段:智能] OR "轻度认知功能损伤"[全部字段:智能] OR "轻度认知功能减退"[全部字段:智能] #9 "衰弱"[全部字段:智能] OR "躯体衰弱"[全部字段:智能] OR "虚弱"[全部字段:智能] OR "脆弱"[全部字段:智能] OR "认知衰弱"[全部字段:智能] #10 #7 OR #8 OR #9 #11 "预测"[不加权:扩展] #12 "预测"[全部字段:智能] OR "预测模型"[全部字段:智能] OR "预测因子"[全部字段:智能] OR "预测因素"[全部字段:智能] OR "危险因素"[全部字段:智能] OR "风险预测"[全部字段:智能] OR "风险评估"[全部字段:智能] OR "列线图"[全部字段:智能] OR "机器学习"[全部字段:智能] #13 #11 OR #12 #14 #3 AND #6 AND #10 AND #13 |

**Supplementary Table S2. Study-specific operational definitions of cognitive frailty and dementia-exclusion methods**

| **Study** | **Cognitive assessment** | **Physical frailty assessment** | **Reported CF definition / threshold** | **Reported dementia-exclusion method** | **Eligibility** | **Outcome-domain judgment** |
| --- | --- | --- | --- | --- | --- | --- |
| Liu 2023 | MoCA | FRAIL scale | Cognitive impairment combined with FRAIL ≥3; CF was defined as occurring in the absence of Alzheimer disease or other dementia. | No clinical Alzheimer disease, other dementia, or neurodegenerative-disease diagnosis. | Retained | Low |
| Jiang 2023 | MoCA + CDR | Fried phenotype | FP ≥3, MoCA 14–25, and CDR 0.5, with no clinical dementia. | CDR and absence of clinical dementia were explicitly required. | Retained | Low |
| Luo 2024 | MMSE + CDR | Fried phenotype | Physical frailty combined with education-adjusted cognitive impairment; CDR 0 or 0.5. | Dementia was excluded in the diagnostic and participant criteria. | Retained | Low |
| Chen et al. 2025 | MoCA | Fried phenotype | Education-adjusted MoCA impairment combined with FP scores of 1–5, including pre-frailty or frailty. | The study conceptually defined CF as occurring in the absence of Alzheimer disease, but the operational procedure used to exclude dementia was not reported. | Retained | Unclear |
| Li et al. 2025 | MMSE | FRAIL scale | Cognitive impairment combined with physical frailty; CF was explicitly defined as excluding Alzheimer disease or other dementia. | Dementia or other neurological diseases were excluded. | Retained | Low |
| Liu et al. 2026 | MoCA | FRAIL scale | FRAIL ≥3 combined with MoCA impairment; a consensus CF definition was applied. | Dementia or neurodegenerative disease was diagnosed by neurologists and documented in medical records. | Retained | Low |
| Wang et al. 2026 | MoCA | Fried phenotype | FP ≥3 combined with education-adjusted MoCA impairment; the conceptual definition specified absence of dementia. | Dementia diagnosed before heart failure was excluded, but the contemporaneous operational procedure was incompletely reported. | Retained | Unclear |
| Gou et al. 2026 | MoCA-BC | FRAIL scale | FRAIL ≥3 combined with MoCA-BC impairment without a dementia diagnosis. | Dementia was explicitly listed as an exclusion criterion. | Retained | Low |

Abbreviations: CDR, Clinical Dementia Rating; CF, cognitive frailty; FP, Fried frailty phenotype; MMSE, Mini-Mental State Examination; MoCA, Montreal Cognitive Assessment; MoCA-BC, Montreal Cognitive Assessment-Basic.

An unclear outcome-domain judgment indicates that the operational dementia-exclusion procedure was incompletely reported and does not mean that dementia was definitely included.

**Supplementary Table S3. Model-level characteristics and performance of the 20 included models**

| **Study / model** | **Role** | **Algorithm / presentation** | **Predictor set** | **Validation and performance dataset** | **AUC (95% CI)** | **Sensitivity** | **Specificity** | **Accuracy / F1** | **Calibration** | **DCA** |
| --- | --- | --- | --- | --- | --- | --- | --- | --- | --- | --- |
| Liu 2023 / LR | Diagnostic / screening | LR nomogram | NYHA class; chewing ability; nutritional status; loneliness; sleep status | Bootstrap internal validation; development-sample and internal-validation estimates | Development sample: 0.804; IV: 0.788 | Development sample: 0.772 | Development sample: 0.703 | NR / NR | H-L test; Brier score; calibration curve | NR |
| Jiang 2023 / LR | Diagnostic / screening | LR nomogram | Age; education; NYHA class; LVEF; sleep duration; nutritional status; depression; intellectual activity; social support | Bootstrap internal validation + external validation | Development sample: 0.854; EV: 0.852 | EV: 0.872 | EV: 0.694 | NR / NR | Calibration curve | NR |
| Luo 2024 / LR | Diagnostic / screening | LR nomogram | Age; education; sleep duration; exercise frequency; NYHA class; CHF duration; cardiac stent history; depression | Bootstrap internal validation + external validation | Development sample: 0.949; EV: 0.799 | Development sample: 0.896; EV: 0.792 | Development sample: 0.876; EV: 0.689 | NR / NR | H-L test; Brier score; calibration curve | NR |
| Chen 2025 / NN | Diagnostic / screening | Neural network | Age; marital status; education; BMI; multimorbidity; nutritional status; medication use; weekly exercise frequency; living status | Random-split internally derived test set | Internally derived test set: 0.820 | 0.700 | NR | 0.803 / 0.778 | NR | NR |
| Chen 2025 / LR | Diagnostic / screening | Logistic regression | Age; marital status; education; BMI; multimorbidity; nutritional status; medication use; weekly exercise frequency; living status | Random-split internally derived test set | Internally derived test set: 0.884 | 0.778 | NR | 0.836 / 0.824 | NR | NR |
| Chen 2025 / NB | Diagnostic / screening | Naive Bayes | Age; marital status; education; BMI; multimorbidity; nutritional status; medication use; weekly exercise frequency; living status | Random-split internally derived test set | Internally derived test set: 0.859 | 0.767 | NR | 0.825 / 0.812 | NR | NR |
| Chen 2025 / LDA | Diagnostic / screening | Linear discriminant analysis | Age; marital status; education; BMI; multimorbidity; nutritional status; medication use; weekly exercise frequency; living status | Random-split internally derived test set | Internally derived test set: 0.883 | 0.778 | NR | 0.847 / 0.833 | NR | NR |
| Chen 2025 / DT | Diagnostic / screening | Decision tree | Age; marital status; education; BMI; multimorbidity; nutritional status; medication use; weekly exercise frequency; living status | Random-split internally derived test set | Internally derived test set: 0.832 | 0.756 | NR | 0.814 / 0.800 | NR | NR |
| Chen 2025 / RF | Diagnostic / screening | Random forest | Age; marital status; education; BMI; multimorbidity; nutritional status; medication use; weekly exercise frequency; living status | Random-split internally derived test set | Internally derived test set: 0.901 | 0.744 | NR | 0.809 / 0.793 | NR | NR |
| Chen 2025 / SVM | Diagnostic / screening | Support vector machine | Age; marital status; education; BMI; multimorbidity; nutritional status; medication use; weekly exercise frequency; living status | Random-split internally derived test set | Internally derived test set: 0.881 | 0.778 | NR | 0.842 / 0.828 | NR | NR |
| Chen 2025 / KNN | Diagnostic / screening | k-nearest neighbors | Age; marital status; education; BMI; multimorbidity; nutritional status; medication use; weekly exercise frequency; living status | Random-split internally derived test set | Internally derived test set: 0.870 | 0.744 | NR | 0.814 / 0.798 | NR | NR |
| Li 2025 / LR | Diagnostic / screening | LR nomogram | Age; regular exercise; ADL; NYHA class; depression | Internal validation + external validation | IV: 0.867; EV: 0.848 (0.790–0.906) | IV: 0.859; EV: 0.839 | IV: 0.760; EV: 0.794 | EV: 0.807 / NR | H-L test; calibration curve | Reported for IV and EV |
| Liu et al. 2026 / LR | Diagnostic / screening | LR nomogram | Age; NYHA class; chewing ability; nutritional status; loneliness; sleep status | External validation; development-sample and EV estimates | Development sample: 0.807 (0.764–0.851); EV: 0.770 (0.667–0.874) | NR | NR | NR / NR | H-L test; calibration curves | Reported for development and EV cohorts |
| Wang 2026 / LR | Diagnostic / screening | LR nomogram | Age; depression; NYHA class; exercise frequency; weekly intellectual activity | No validation; development-sample estimate only | Development sample: 0.846 (0.797–0.895) | 0.779 | 0.800 | NR / NR | Goodness-of-fit test; calibration curve | NR |
| Gou 2026 / CatBoost | Diagnostic / screening | Categorical boosting | Age; sex; education; marital status; CHF duration; CHF hospitalization history; NYHA class; malnutrition; ADL; sleep disturbance; depression | Random split + 5-fold cross-validation + bootstrap; internally derived test set | Internally derived test set: 0.916 (0.877–0.952) | 0.9221 | 0.8050 | 0.8476 / 0.8150 | Brier score: 0.113 | Reported |
| Gou 2026 / LR | Diagnostic / screening | Logistic regression | Age; sex; education; marital status; CHF duration; CHF hospitalization history; NYHA class; malnutrition; ADL; sleep disturbance; depression | Random split + 5-fold cross-validation + bootstrap; internally derived test set | Internally derived test set: 0.890 (0.844–0.935) | 0.8790 | 0.7783 | 0.8151 / 0.7767 | Brier score: 0.131 | Reported |
| Gou 2026 / RF | Diagnostic / screening | Random forest | Age; sex; education; marital status; CHF duration; CHF hospitalization history; NYHA class; malnutrition; ADL; sleep disturbance; depression | Random split + 5-fold cross-validation + bootstrap; internally derived test set | Internally derived test set: 0.901 (0.858–0.937) | 0.8815 | 0.7826 | 0.8191 / 0.7811 | Brier score: 0.127 | Reported |
| Gou 2026 / SVM | Diagnostic / screening | Support vector machine | Age; sex; education; marital status; CHF duration; CHF hospitalization history; NYHA class; malnutrition; ADL; sleep disturbance; depression | Random split + 5-fold cross-validation + bootstrap; internally derived test set | Internally derived test set: 0.905 (0.858–0.942) | 0.8657 | 0.8254 | 0.8403 / 0.7978 | Brier score: 0.121 | Reported |
| Gou 2026 / MLP | Diagnostic / screening | Multilayer perceptron | Age; sex; education; marital status; CHF duration; CHF hospitalization history; NYHA class; malnutrition; ADL; sleep disturbance; depression | Random split + 5-fold cross-validation + bootstrap; internally derived test set | Internally derived test set: 0.900 (0.854–0.936) | 0.8939 | 0.7732 | 0.8177 / 0.7827 | Brier score: 0.127 | Reported |
| Gou 2026 / XGBoost | Diagnostic / screening | Extreme gradient boosting | Age; sex; education; marital status; CHF duration; CHF hospitalization history; NYHA class; malnutrition; ADL; sleep disturbance; depression | Random split + 5-fold cross-validation + bootstrap; internally derived test set | Internally derived test set: 0.892 (0.838–0.934) | 0.8731 | 0.7873 | 0.8186 / 0.7778 | Brier score: 0.130 | Reported |

Abbreviations: ADL, activities of daily living; AUC, area under the receiver operating characteristic curve; BMI, body mass index; CatBoost, categorical boosting; CHF, chronic heart failure; DCA, decision-curve analysis; DT, decision tree; EV, external validation; F1, F1 score; H-L, Hosmer–Lemeshow; IV, internal validation; KNN, k-nearest neighbors; LDA, linear discriminant analysis; LR, logistic regression; LVEF, left ventricular ejection fraction; MLP, multilayer perceptron; NB, naive Bayes; NN, neural network; NR, not reported; NYHA, New York Heart Association; RF, random forest; SVM, support vector machine; XGBoost, extreme gradient boosting.

95% confidence intervals are presented where reported in the primary studies. For Chen 2025, recall was treated as sensitivity. Random-split test sets derived from the same source cohort were classified as internal validation rather than external validation. Development-sample, internal-validation, and external-validation estimates were not treated as directly comparable.

**Supplementary Table S4. Study-specific PROBAST judgments and rationales**

| **Study** | **Participants** | **Predictors** | **Outcome** | **Analysis** | **Overall risk of bias** | **Overall applicability** |
| --- | --- | --- | --- | --- | --- | --- |
| Liu 2023 | Low: the hospitalized older-adult CHF population was appropriate for the review question. | Unclear: predictor assessment without knowledge of outcome status was not clearly reported, and some nutritional or activity-related variables were conceptually close to FRAIL components. | Low: standardized cognitive and frailty assessments were used, and Alzheimer disease, other dementia, or neurodegenerative disease was excluded. | High: data-driven variable screening, direct deletion of missing data, limited effective sample size, and inadequate control of overfitting and optimism. | High | Low concern |
| Jiang 2023 | Low: the multicenter hospitalized CHF sample was appropriate. | Unclear: predictor assessment without knowledge of outcome status was not clearly reported, and some nutritional or activity-related variables were conceptually close to Fried phenotype components. | Low: MoCA, the Fried phenotype, CDR, and explicit absence of clinical dementia were used. | High: direct deletion of missing data and residual overfitting/optimism concerns despite bootstrap internal validation and external validation. | High | Low concern |
| Luo 2024 | Low: the multicenter hospitalized CHF sample was appropriate. | High: exercise frequency conceptually overlapped with the low-physical-activity component of the Fried phenotype, and predictor blinding was unclear. | Low: education-adjusted cognitive assessment, the Fried phenotype, and CDR-based dementia exclusion were reported. | High: univariate screening, direct deletion of missing data, and incomplete adjustment for overfitting and optimism. | High | Low concern |
| Chen 2025 | Low: the multicenter hospitalized CHF sample was appropriate. | High: several predictors were measured concurrently and included activity-related constructs close to the physical frailty outcome; predictor assessment without knowledge of outcome status was unclear. | Unclear: the study conceptually required absence of Alzheimer disease, but the operational procedure used to exclude dementia was not reported. | High: random-split internal validation, multiple algorithm comparisons, no reported calibration, and limited control of optimism. | High | Low concern |
| Li 2025 | Low: the hospitalized CHF sample was appropriate. | High: regular exercise and ADL conceptually overlapped with activity, ambulation, or endurance components of physical frailty. | Low: standardized cognitive and frailty criteria were used, with explicit exclusion of dementia or neurological disease. | High: univariate/stepwise selection and incomplete accounting for overfitting despite internal and external validation. | High | Low concern |
| Liu et al. 2026 | Low: the multicenter hospitalized CHF sample was appropriate. | Unclear: predictor assessment without knowledge of outcome status and some predictor definitions were insufficiently reported; nutritional and functional constructs were close to the frailty outcome. | Low: standardized assessments were used, and neurologists documented dementia or neurodegenerative-disease exclusion. | High: univariate screening, direct deletion of missing data, and incomplete quantification of optimism despite external validation. | High | Low concern |
| Wang 2026 | High: retrospective inclusion of patients with complete clinical data may have introduced selection bias. | High: exercise frequency conceptually overlapped with the Fried low-physical-activity component, and predictor blinding was unclear. | Unclear: pre-existing dementia was excluded, but the contemporaneous operational procedure for dementia exclusion was incompletely reported. | High: complete-case selection, no internal or external validation, and inadequate assessment of overfitting and optimism. | High | Low concern |
| Gou 2026 | Low: the hospitalized CHF sample was appropriate. | High: ADL, malnutrition, and activity-related variables conceptually overlapped with FRAIL components. | Low: standardized cognitive and frailty assessments were used, and dementia was explicitly excluded. | High: low EPV, a large candidate-predictor set, internal validation based on random splitting/cross-validation/bootstrap without external validation, and residual overfitting concerns. | High | Low concern |

Abbreviations: ADL, activities of daily living; CDR, Clinical Dementia Rating; CF, cognitive frailty; CHF, chronic heart failure; EPV, events per variable; MoCA, Montreal Cognitive Assessment; PROBAST, Prediction Model Risk of Bias Assessment Tool.
